# In-cell NMR as a sensitive tool to monitor physiological condition of *Escherichia coli*

**DOI:** 10.1038/s41598-020-59076-2

**Published:** 2020-02-12

**Authors:** Toshihiko Sugiki, Yoshihiro Yamaguchi, Toshimichi Fujiwara, Masayori Inouye, Yutaka Ito, Chojiro Kojima

**Affiliations:** 1 Institute for Protein Research, Osaka University, 3-2 Yamadaoka, Suita, Osaka 565-0871 Japan; 2 The OCU Advanced Research Institute for Natural Science and Technology, Osaka City University, 3-3-138 Sugimoto, Sumiyoshi, Osaka 558-8585 Japan; 3 Department of Biochemistry and Molecular Biology, Rutgers University, 675 Hoes Lane, Piscataway, NJ 08854 USA; 4 Department of Chemistry, Graduate School of Science, Tokyo Metropolitan University, 1-1 Minami-Osawa, Hachioji, Tokyo 192-0397 Japan; 5 Graduate School of Engineering, Yokohama National University, 79-5 Tokiwadai, Hodogaya-ku, Yokohama 240-8501 Japan

**Keywords:** NMR spectroscopy, Expression systems

## Abstract

The in-cell NMR technique offers significant insights into the structure and function of heterologous proteins in the physiological intracellular environment at an atomic resolution. *Escherichia coli* (*E*. *coli*) is one of the most widely used host cells for heterologous protein expression in structural biological studies as well as for in-cell NMR studies to investigate fundamental structural characteristics and the physiochemistry of certain proteins and their intermolecular interactions under physiological conditions. However, in many cases, it is not easy to obtain well-resolved in-cell NMR spectra because the detectability and resolution of these spectra are significantly influenced by intracellular factors such as nonspecific intermolecular interactions. In this study, we re-examined the experimental parameters of *E*. *coli* in-cell NMR and found that the detectability and resolution of the NMR spectra clearly depended on the growth phase of the host cells. Furthermore, the detectability and resolution of the *E*. *coli* in-cell NMR spectra correlated with the soluble fraction amounts of the expressed target protein. These results indicate that the *E*. *coli* in-cell NMR spectrum of a target protein is a useful tool for monitoring the intracellular conditions of the host cell and for establishing the appropriate cultivation conditions for protein overexpression.

## Introduction

Solution NMR spectroscopy is nondestructive and is therefore widely used as a tool for the *in vivo* observation of metabolites and metal ions in living cells, for which it is ideal^1^. For example, *in vivo* quantitative, real-time monitoring of the dynamics of cellular sodium ions or ATP/ADP has been established by measuring the NMR spectra of ^23^Na or ^31^P nuclei, respectively^1^. Recently, *in vivo* NMR techniques have not only been applied to small biomolecules but have also been developed as in-cell NMR to study the structure and dynamics of heterologous proteins overexpressed in host cells.

In living cells, most proteins exert their biological function in an extremely crowded environment due to the high concentration of macromolecules (which can reach 400 g/L)^2,3^, and this can affect their conformation, stability, dynamics, and function^4–7^. The in-cell NMR technique can be used in the physiological intracellular environment to gain significant knowledge about the structural and functional mechanisms of heterologous proteins at an atomic resolution^8–14^.

*Escherichia coli* (*E*. *coli*) is one of the most widely used host cells for the overexpression of heterologous proteins, as well as for in-cell protein NMR studies to elucidate fundamental structural and physicochemical characteristics of proteins and their intermolecular interaction modes under physiological conditions^8–14^. However, in many cases it is not straightforward to record well-resolved *E*. *coli* in-cell NMR spectra for proteins as their detectability and resolution can be negatively influenced by many factors in the living host cells, such as nonspecific intermolecular associations. Conversely, this means that *E*. *coli* in-cell NMR spectra for proteins can sensitively reflect the intracellular physiological environment or phenomena involving protein dynamics.

Given that *E*. *coli* is the most widely utilized bioreactor for the overexpression of heterologous proteins, it is possible that the *E*. *coli* in-cell NMR spectrum of a recombinant protein would reflect the physiological conditions in a living host cell during the overexpression of that protein. In this study, we comprehensively re-examined the experimental parameters of *E*. *coli* in-cell NMR and investigated the types of information that would be reflected in the in-cell NMR spectrum of a recombinant protein.

## Results and Discussions

### The detectability and resolution of *E*. *coli* in-cell NMR spectra were significantly influenced by experimental parameters

It is widely accepted that the detectability and resolution of *E*. *coli* in-cell NMR spectra are influenced by several experimental parameters, such as the host cell cultivation conditions (e.g., the process or media composition of the subculture)^8,15^ and observation conditions (e.g., the final cell density in the NMR sample tube)^16^. In this study, we first re-examined the experimental parameters to elucidate the critical factors that could result in detectable and reproducible *E*. *coli* in-cell NMR measurements. Four experimental parameters of *E*. *coli* in-cell NMR were selected: the concentration of D_2_O in an NMR sample for ^2^H locking of the static magnetic field; the final cell density in the NMR sample tube; the final concentration of isopropyl β-d-1-thiogalactopyranoside (IPTG); and the cell density of the culture media (OD_600_ value for each stage of the growth curve) at the time of the addition of IPTG.

A previous report by Serber and colleagues indicated that a higher final cell density in an NMR sample tube gave broader, lower-resolution *E*. *coli* in-cell NMR spectra with lower sensitivity for the ^2^H lock signal^15,16^. The same tendency was observed in the present study, that is, a higher concentration of D_2_O and a lower cell density in the NMR sample can result in improved resolution of the *E*. *coli* in-cell NMR spectrum (Supplemental Fig. S1A,B). The detectability and resolution of an *E*. *coli* in-cell NMR spectrum do not depend on the IPTG dose (Supplemental Fig. S1C). The detectability and resolution of *E*. *coli* in-cell NMR spectra of GB1 drastically depended on the OD_600_ value at the time of the addition of IPTG, and this parameter was the most critical to improve in-cell NMR spectra (Supplemental Fig. S1).

To confirm whether the NMR spectra of the target protein definitely originated within the living host cells, the NMR spectrum of *E*. *coli* suspension supernatant was measured immediately after the in-cell NMR measurement. No NMR signal was detected (Supplemental Fig. S2); thus, the improvement in the resolution of the *E*. *coli* in-cell NMR spectrum was not due to leakage of the target protein into the extracellular solvent in the NMR sample tube. In addition, we confirmed that in-cell NMR signals of target protein was not from the leakage expression of pET expression system (Supplemental Fig. S3).

### The *E*. *coli* in-cell NMR spectrum for GB1 drastically depended on the stage of the growth curve

Because the OD_600_ value at the time of the addition of IPTG was a critical factor, in this section, the correlation between this and the detectability and resolution of the associated *E*. *coli* in-cell NMR spectrum was investigated in detail. Various *E*. *coli* cell suspension samples were obtained at all stages of the growth curve. Overexpression of the heterologous target protein was induced by IPTG at various OD_600_ values. In-cell NMR spectra for all samples were measured.

**Figure 1:**
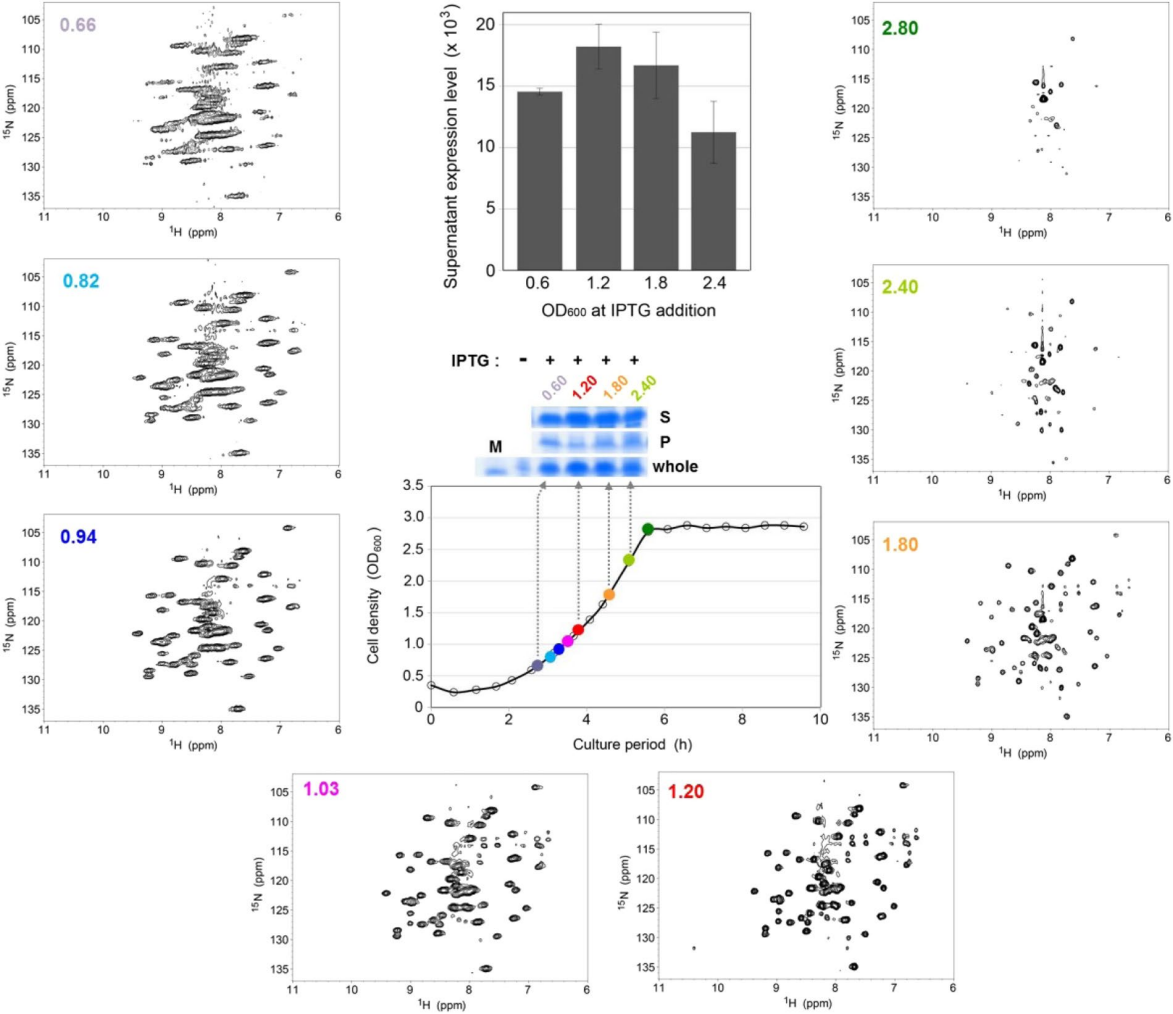
A series of *E*. *coli* in-cell 2D ^1^H-^15^N TROSY-HSQC NMR spectra for [*U*-^15^N]GB1. For the overexpression of the [*U*-^15^N] GB1 proteins, IPTG was added at various cell density (OD_600_) values and cell cultivation was continued for a further 3 h. The growth curve for the pET30/GB1 transformants of the *E*. *coli* BL21(DE3) strain is shown in the center panel. Each OD_600_ value at the time of the addition of IPTG to the individual in-cell NMR spectrum is indicated at the top left corner of the NMR spectrum, colored according to the time of the addition of IPTG, which is indicated on the growth curve as colored dots. Protein expression levels were confirmed by performing SDS–PAGE and Coomassie Brilliant Blue staining of the gel (above the panel of growth curve). “Whole” denoted in the center panel indicates the total protein expression level, S and P indicate the protein expression levels in the soluble and insoluble fractions, respectively, and M indicates the molecular weight marker. The associated entire SDS–PAGE is shown in Supplemental Fig. S13. Protein expression levels of supernatant shown in the SDS–PAGE are quantified by ImageJ analysis (top-center panel). The error bars on the graph are based on the results from three independent experiments.

*E*. *coli* in-cell NMR signals were severely line-broadened when the OD_600_ value at the time of the addition of IPTG was 0.60, corresponding to the late lag to early log phase (Fig. 1 and Supplemental Fig. S4). The signals became narrower when the OD_600_ value was around 1.00, corresponding to the early to middle log phase, and the signal intensity decreased when the OD_600_ value was larger than 1.80, corresponding to the middle log to the stationary phase (Fig. 1). At the middle log to stationary phase, several unidentified strong signals appeared around 8 ppm (^1^H) and 115–120 ppm (^15^N) chemical shifts in the associated *E*. *coli* in-cell NMR spectra (Fig. 1). Thus, the detectability and resolution of the in-cell NMR spectra depended to a great extent on the stage of the growth curve (Fig. 1).

To confirm that the NMR spectra of the target protein definitely originated within the living host cells, the NMR spectra of *E*. *coli* lysate, in which overexpression of the target protein were induced by adding IPTG at various OD_600_ values in a similar manner as described above, were measured. As a result, signal line-width of all NMR spectra were remarkably improved (Supplemental Fig. S5). Its results support that the in-cell NMR spectra (Fig. 1), which shows host *E*. *coli* cell growth phase-dependent alterations of the detectability and resolution, were certainly originated in the living host cells.

Several other proteins, TTHA0227, TTHA0814, TTHA1718, hCaM and GFP, were tested to investigate whether the dependency of the *E*. *coli* in-cell NMR spectrum on the stage of the growth phase was not a GB1-specific phenomenon. The GB1, TTHA1718 and hCaM proteins are widely utilized for *E*. *coli* in-cell NMR experiments because they provide detectable *E*. *coli* in-cell NMR spectra^15–17^. However, there has been no report describing the in-cell NMR measurement of GFP, even though this is widely utilized as an analytical tool to visualize the intracellular localization of target proteins in a cell.

**Figure 2:**
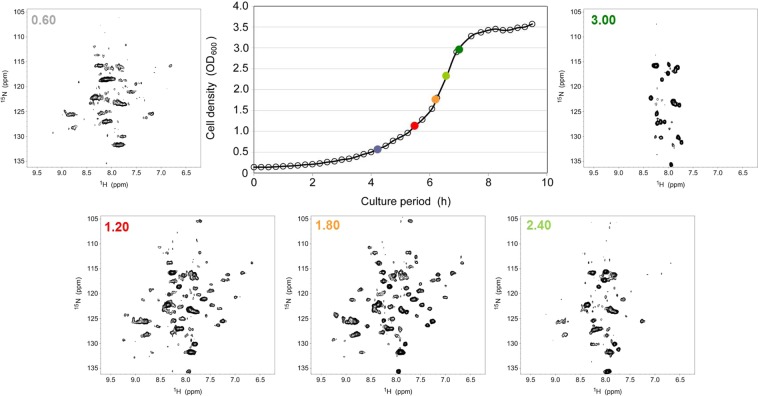
A series of *E*. *coli* in-cell 2D ^1^H-^15^N TROSY-HSQC NMR spectra for [*U*-^15^N]TTHA1718 were measured for various cell density (OD_600_) values at the time of the addition of IPTG. The growth curve for the pET11/TTHA1718 transformants of the *E*. *coli* BL21(DE3) strain is shown in the upper right panel. The OD_600_ value at the time of the addition of IPTG to the individual in-cell NMR spectrum is denoted in the top left corner of each NMR spectrum.

As shown in Fig. 2 and Supplemental Fig. S6, TTHA1718 showed similar tendencies to those of GB1, that is, substantial line-broadening at the late lag to early log phase, narrowing at the early to middle log phase, and a decrease at the middle log to stationary phase. Other TTHAs, hCaM and GFP did not show clear in-cell NMR signals (Supplemental Figs. S7–S11). For most samples, several unidentified strong signals appeared at similar positions at the middle log to stationary phase as with GB1. Indeed, superposition of the 2D ^1^H-^15^N HSQC spectra of GB1, hCaM, and GFP showed that the chemical shift values of these unidentified strong signals completely matched across the three spectra (Supplemental Fig. S12)^18–20^.

It was previously reported that bacterial cell lysate containing over-expressed heterologous proteins can be contaminated with water-soluble carbohydrates such as cyclic enterobacterial common antigen (ECA_CYC_)^21,22^. Interestingly, the previous reports showed that the amount of the carbohydrate contaminants were increased depending on the cell culture conditions, such as length of cell culture period after induction of protein over-expression and composition of the culture media^22^. Our study demonstrated that the unidentified strong signals appeared depending on the length of cell culture period, more specifically the “before” induction of protein over-expression (Figs. 1, 2, and Supplemental Figs. S7–S10).

Our NMR study showed that the ^1^H-^15^N chemical shifts of the unidentified strong signals were extremely identical to that of *E*. *coli* ECA_CYC_ (Figs. 1, 2, and Supplemental Figs. S7–S12). On the other hand, our further NMR experiments also demonstrated another possibility that the unidentified strong signals may be derived from fragments of cell wall peptidoglycan from the *E*. *coli* host cell (Supplemental Fig. S13).

These results indicate that the unidentified strong signals are not derived from the over-expressed heterologous proteins, but are derived from components of the host cells. Moreover, the results support the dependency of *E*. *coli* in-cell NMR spectrum on “stage of the growth curve” as a universal phenomenon regardless of the kind of over-expressed heterologous protein. In addition, they suggest that *E*. *coli* in-cell NMR spectra can sensitively reflect cell culture conditions during protein over-expression as further discussed below.

### The *E*. *coli* in-cell NMR spectra depended on the yield of soluble and healthy protein

To identify the origin of the dependency of *E*. *coli* in-cell NMR spectra on the stage of the growth curve, we investigated the relationship between the spectra and the amount of soluble fraction of the overexpressed protein. As shown in Fig. 1 and Supplemental Figs. S14–S15, the soluble fraction of the overexpressed protein per medium reached its maximum when the protein overexpression was induced at OD_600_ = 1.20, and the best *E*. *coli* in-cell NMR spectrum (among those of OD_600_ = 0.6, 1.2, 1.8, and 2.4) was obtained at OD_600_ = 1.20 (Fig. 1). It is notable that the expression level of the insoluble fraction was slightly decreased when IPTG was added at OD_600_ = 1.20 (Fig. 1).

These results indicate that most efficient condition to yield soluble and healthy recombinant protein and give the best *E*. *coli* in-cell NMR spectra is to induce the overexpression of the target protein during the early to middle log phase. This suggests that the origin of the dependency of *E*. *coli* in-cell NMR spectra on the stage of the growth curve of the target protein arises from the yield of soluble and healthy recombinant proteins, making this a good tool for monitoring the best conditions for heterologous protein overexpression.

Two different expression systems, pET^23–25^ and pCold^26^, were tested to investigate whether *E*. *coli* in-cell NMR spectra were dependent on the expression system. The pCold expression system utilized cold-shock responses for heterologous protein overexpression and improved the expression level of soluble and healthy recombinant protein, suggesting that the intracellular physiological environment differed between the pET and pCold systems.

**Figure 3:**
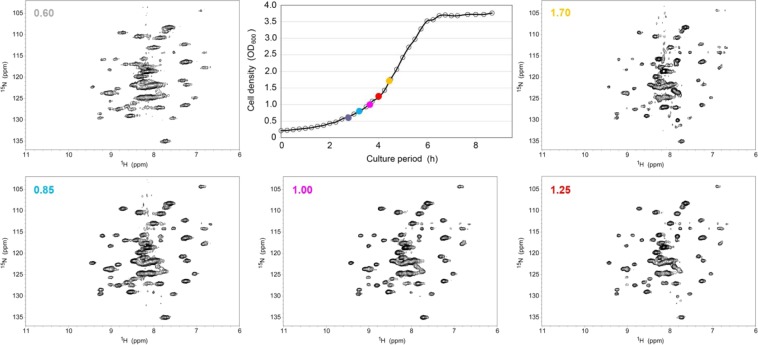
A series of *E*. *coli* in-cell 2D ^1^H-^15^N TROSY-HSQC NMR spectra of [*U*-^15^N]GB1, with the protein overexpressed by using the pCold expression system, were measured for various cell density (OD_600_) values at the time of addition of IPTG. The growth curve of the pCold I/GB1 transformants of the *E*. *coli* BL21(DE3) strain is shown on the upper-right panel. For overexpression of the [*U*-^15^N]GB1 proteins, IPTG was added at various OD_600_ values (0.60, 0.85, 1.00, 1.25, and 1.70), and each *E*. *coli* in-cell NMR spectrum was measured after a further 24 h of cell cultivation. The each OD_600_ value at the time of the addition of IPTG to the individual in-cell NMR spectrum is indicated at the top left corner of each spectrum.

As shown in Fig. 3 and Supplemental Fig. S16, the pCold expression system showed much less dependency of the NMR spectrum on the growth curve than did the pET system (Fig. 1 and Supplemental Fig. S4). In the pCold system, the *E*. *coli* in-cell NMR spectra showed relatively higher resolution during the late lag to early log phase (Supplemental Figs. S4 and S16). This result is consistent with the difference in the potential soluble protein overexpression; that is, the pCold system is less sensitive to the induction timing of protein overexpression than the pET system.

The pCold system showed a similar tendency as the pET system, i.e., line-broadening at the late lag to early log phase, narrowing at the early to middle log phase, and a decrease at middle log to stationary phase (Figs. 1 and 3). Several unidentified strong signals also appeared at similar positions during the middle log to stationary phase, just as with the pET system. These results suggest that dependency of the host cell growth phase at the starting point of protein overexpression induction on the detectability and resolution of *E*. *coli* in-cell NMR spectra is not a phenomenon that is specific to the expression system.

### The *E*. *coli* in-cell NMR spectra depend on the induction period

**Figure 4:**
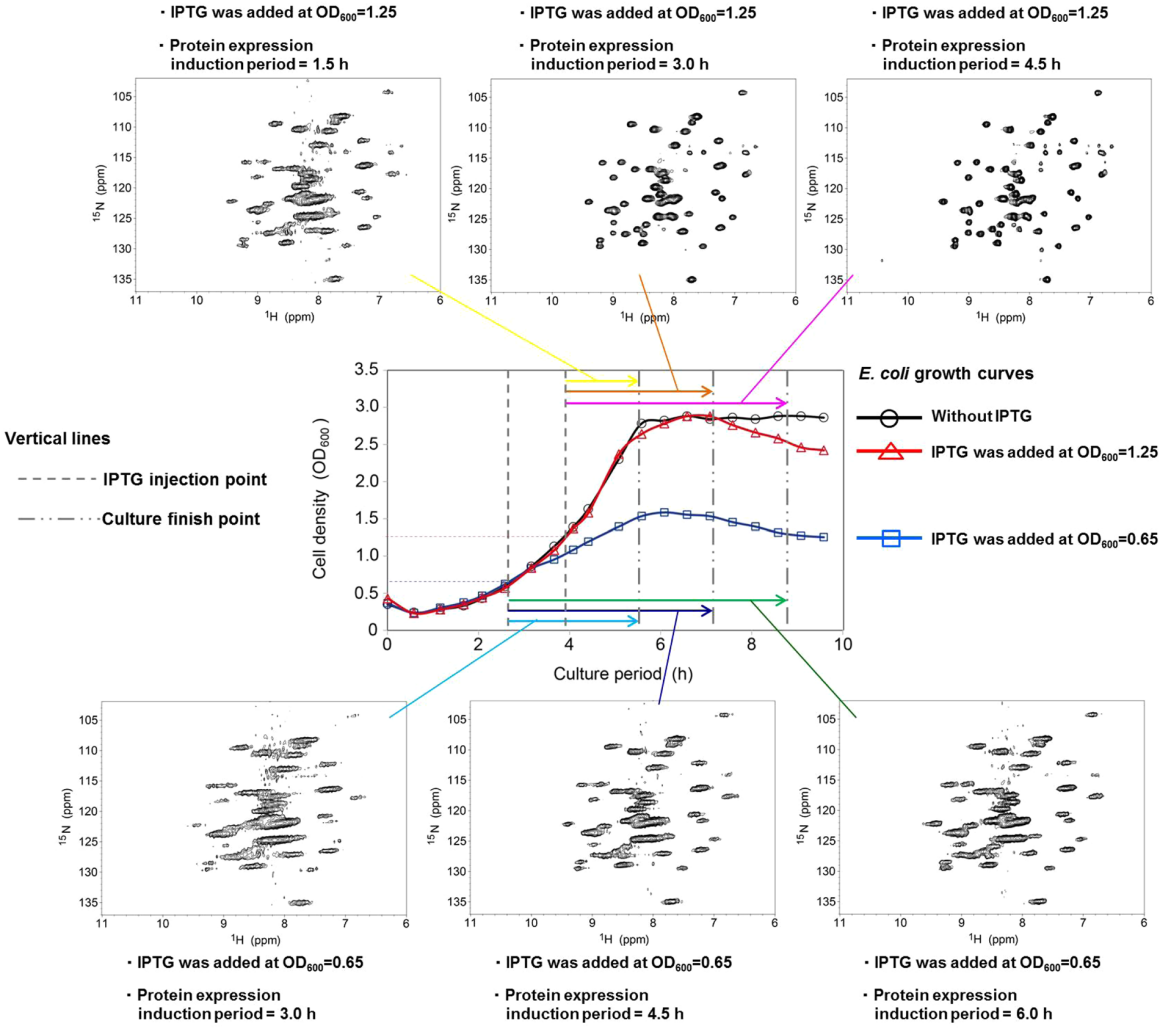
Growth phase-dependent alteration of *E*. *coli* in-cell NMR spectra. The *E*. *coli* NMR spectra were recorded for various protein expression induction periods and/or finishing times. The plotted black open circles, blue open squares, and red open triangles in the center panel are the growth curves for the pET30/GB1 transformants of the *E*. *coli* BL21(DE3) strain in the cases of no added IPTG, and with IPTG addition when the OD_600_ value was 0.65 or 1.25, respectively. The six colored arrows indicate the time of the addition of IPTG and protein expression induction period (the finishing point for the cell cultivation) for each *E*. *coli* in-cell NMR spectrum.

As described earlier, the detectability and resolution of the *E*. *coli* in-cell NMR spectra depended on the stage of the growth curve. Serber and colleagues reported that the detectability and resolution of the *E*. *coli* in-cell NMR spectra depended on the duration of the induction period^15^. Thus, results of our study demonstrated that the detectability and resolution of *E*. *coli* in-cell NMR spectra may depend on both the stage of the growth curve and the duration of the induction period. To evaluate the significance of these two factors, *E*. *coli* in-cell NMR spectra were measured at various durations of the induction period and at two different stages of the growth curve, namely OD_600_ values ~0.65 and 1.25, during the late lag to early log phase and the middle log phase, respectively. At both stages, OD_600_ ~ 0.65 and 1.25, the resolution was better for the *E*. *coli* in-cell NMR spectrum with the longer induction period (Fig. 4).

*E*. *coli* in-cell NMR spectra with the same finishing point for the induction but with different starting points and induction periods were compared. The spectrum associated with induction during the late lag to early log phase (OD_600_ value ~0.65) with a 4.5-h induction period had the same finishing point of induction as those induced in the middle log (OD_600_ value ~1.25) and stationary (OD_600_ value ~2.6) phases with 3.0- and 1.5-h induction periods, respectively (Fig. 4 and Supplemental Fig. S17). The detectability and resolution of the *E*. *coli* in-cell NMR spectra clearly depended on the starting point of induction, and the best point for this was the middle log phase (OD_600_ value ~1.25), as well as described in the Results section. These results demonstrate that the detectability and resolution of *E*. *coli* in-cell NMR spectra depended on both the stage of the growth curve and the duration of the induction period. Strictly, it was the starting point of induction, not the duration of induction, that was the critical determinant of the detectability and resolution of *E*. *coli* in-cell NMR spectrum.

### Physiological meaning

The results of this study showed that the starting point of induction is the critical determinant of the detectability and resolution of *E*. *coli* in-cell NMR spectra. In addition, we have proposed that the origin of the dependency of these spectra on the stage of the growth curve of the target protein arises from the yield of soluble and healthy recombinant proteins. However, the physiological meaning of this is not clear.

To establish the physiological meaning of the starting point of induction, we analyzed the *E*. *coli* growth curves for the cases where IPTG was added when OD_600_ was ~0.65 or 1.25. In these cases, the OD_600_ values finally reached 1.5 and 2.6 at the stationary phase, respectively. The OD_600_ value 2.6 was identical to the case of no induction; that is, when the starting point of induction was the middle log phase, the *E*. *coli* cell was able to grow healthily similar to the case where there was no induction. These results suggest that the detectability and resolution of *E*. *coli* in-cell NMR spectrum can provide information about the intra-cellular physiological conditions of the *E*. *coli* host cells. It should be noted that each growth curve stage is from the mixture of all phases in the life cycle of the *E*. *coli* host cell, as the duplication time of the cell ~20 min is significantly shorter than the time range of both the growth curve ~6 hours and the induction time of the protein overexpression ~2–6 hours. Therefore, any in-cell NMR spectra represent the mixture of all phases in the life cycle of the *E*. *coli* host cell.

In this study, we showed that the detectability and resolution of *E*. *coli* in-cell NMR spectra depended on the growth curve stage of the host cells, and in particular on the starting point of the induction of the target protein overexpression. The spectra correlated with the soluble expression level of the target protein, and the origin of the dependency of the spectra on the stage of the growth curve of the target protein was expected to arise from the yield of soluble and healthy recombinant proteins. When the starting point of induction was the middle log phase, the *E*. *coli* cell was able to grow healthily, similar to the no induction case, and this gave the best spectrum. Thus, the *E*. *coli* in-cell NMR spectrum can provide information about the intracellular physiological conditions in the *E*. *coli* host cells as ensemble average, and could be utilized to monitor soluble and healthy protein production.

## Methods

All chemicals were purchased from Wako Chemicals (Osaka, Japan) and Nacalai Tesque, Inc.(Kyoto, Japan) unless specifically noted, except for the isotope-enriched amino acids, which were purchased from Cambridge Isotope Laboratories.

### Protein expression

The pET expression plasmid containing cDNA coding GB1 (the T2Q mutant, pI = 4.5, whose amino acid sequence is MQYKLILNGKTLKGETTTEAV DAATAEKVFKQYANDNGVDGEWTYDDATKTFTVTE), *thermus thermophilus* HB8 (TTHA1718) and its homologues (TTHA0227, TTHA0814), human calmodulin (hCaM), and green fluorescent protein (GFP) were individually incorporated into the *E*. *coli* BL21(DE3) strain (New England Biolabs, MA, USA). A fresh single colony of the BL21(DE3) transformants was picked and cultivated in 15 mL LB medium in a 50 mL Falcon tube at 37 °C overnight (ca. 12 h). The whole cell suspension was then centrifuged at 2,000 *g* for 10 min at room temperature, and its supernatant was eliminated. The cell pellets were re-suspended in 150 mL of fresh M9 minimal media, which contained ^15^NH_4_Cl (1 g/L) and isotopically unlabeled d-glucose (4 g/L) as the sole nitrogen and carbon sources, respectively; this was aseptically prepared in a 300-mL baffled Erlenmeyer flask and cultured at 37 °C with horizontally rotational shaking at 200 rpm. When the cell density (OD_600_; optical density at 600 nm) values reached the desired value, IPTG was added into the medium to achieve a final concentration of 1 mM, and cell cultivation was continued for a further period (typically 3 h). After the cell cultivation finished, the cell suspension was centrifuged at 2,000 *g* for 10 min at room temperature, and the required amount of cell pellets (typically ca. 0.55 g wet cell weight) was gently re-suspended with ~500 μL of fresh M9 minimal media containing 10% [v/v] D_2_O. The cell suspension was then gently transferred into a 5-mm diameter glass tube.

### SDS–PAGE

Fractionation of the cell lysate into soluble and insoluble components (the supernatant and precipitate, respectively) was performed as follows. First, 5 mL of cell suspension was centrifuged at 2,000 *g* for 10 min at room temperature, and the cell pellet was washed gently once using phosphate buffered saline (PBS). The cell pellet was gently re-suspended using 1 mL of fresh PBS, and the cells were disrupted with sonication. Following centrifugation at 17,000 *g* for 10 min at 4 °C, 75 μL of the supernatant was retrieved and was mixed well with 25 μL of 4 × loading buffer for SDS–PAGE and 1 μL of 2-mercaptoethanol. The residual pellet (cell debris) was re-suspended with 600 μL of PBS and was mixed well with 200 μL of 4 × loading buffer for SDS–PAGE and 8 μL of 2-mercaptoethanol. SDS–PAGE was applied to 10 μL aliquots of these soluble and insoluble fractions; the SDS–PAGE gel was then stained using Coomassie Brilliant Blue (CBB) and the area of the band of the target protein was quantified by using ImageJ software.

### NMR spectroscopy

All of the NMR spectra were recorded on either a Bruker Ascend 500 MHz or an AVANCE 600 MHz equipped with a cryogenic BBO or QCI-P probe, respectively, at a sample temperature of 25 °C. Two-dimensional (2D) ^1^H-^15^N BEST-TROSY NMR spectra, with 1024 data points in the F2 (^1^H) dimension and 96 increments with 16 ppm and 36 ppm spectral width in the F2 and F1 (^15^N) dimensions, respectively, and glycine-selective ^1^H-^15^N HSQC with MUSIC^27,28^ were recorded with 32 scans. The NMR data were processed and analyzed using programs NMRPipe^29^ and Sparky (T. D. Goddard and D. G. Kneller, SPARKY 3, University of California, San Francisco), respectively.

## Supplementary information

Supplemental Data.
