# Supplementary material for: In-cell NMR as a sensitive tool to monitor physiological condition of Escherichia coli
Source: Sci Rep. 2020 Feb 12;10:2466. doi: 10.1038/s41598-020-59076-2 (PMC7015911; doi:10.1038/s41598-020-59076-2)
Supplement: Supplementary file 1 — Supplemental Data. [file 41598_2020_59076_MOESM1_ESM.pdf]

Supplemental Data

In-cell NMR as a sensitive tool to monitor physiological condition of *Escherichia coli*

Toshihiko Sugiki[1], Yoshihiro Yamaguchi[2], Toshimichi Fujiwara[1], Masayori Inouye[3], Yutaka Ito[4], and Chojiro Kojima[1,5]

[1]Institute for Protein Research, Osaka University, 3-2 Yamadaoka, Suita, Osaka 565-0871, Japan

[2]The OCU Advanced Research Institute for Natural Science and Technology, Osaka City University, 3-3-138 Sugimoto, Sumiyoshi, Osaka 558-8585, Japan

[3]Department of Biochemistry and Molecular Biology, Rutgers University, 675 Hoes Lane, Piscataway, NJ 08854

[4]Department of Chemistry, Graduate school of Science, Tokyo Metropolitan University, 1-1 Minami-Osawa, Hachioji, Tokyo 192-0397, Japan

[5]Graduate School of Engineering, Yokohama National University, 79-5 Tokiwadai, Hodogaya-ku, Yokohama 240-8501, Japan

Correspondence should be addressed to C.K. (email: kojima-chojiro-xk@ynu.ac.jp)

E. coli in-cell NMR tips demonstrated in this study

When higher-quality *E. coli* in-cell NMR spectra was measured, the following phenomena were seen prior to the start of the in-cell NMR measurements in many cases:

(1) The intensity of the ^{2}H lock signal of $^{2}H_2O$ was sufficiently high, indicating that locking of the magnetic field can be accomplished smoothly. Conversely, when the intensity of the ^{2}H lock signal of $^{2}H_2O$ was weak, the locking was prone to being aborted and the quality of the in-cell NMR spectra was low even if locking of the static magnetic field was forced to work by increasing ^{2}H lock power. Serber and colleagues have reported that too great a cell density in an NMR sample tube stalled the sensitivity of the ^{2}H lock signal and led to a loss of sensitivity of shimming (Serber et al. 2001a, b; Reckel et al. 2007). In addition to this insight of Serber's, our results indicated that attenuation of the ^{2}H lock signal is a general phenomenon seen not only when there is too great a cell density in the NMR sample tube but also when the quality of the in-cell NMR spectra is low.

(2) Automatic shimming programs (such as TopShim, provided by Bruker (Fällanden, Switzerland)) work successfully without being aborted, and the standard deviation of the final B0 value (which is an indicator of the homogeneity of the static magnetic field through the sample, measured by TopShim) after running the automatic shimming could be less than 0.5 Hz. When the automatic shimming program was aborted or the standard deviation of the final B0 value after automatic shimming was not improved above around 1–2 Hz, it was hardly worth continuing to set up the in-cell NMR measurement of the current sample. The abortion of, or poor results from,

automatic shimming has been attributed to the weak intensity of the ^{2}H lock signal, as described above.

The assignment of *E. coli* in-cell NMR-specific unknown signals and the evaluation of their origin molecules.

We attempted to identify the origin of identified signals because assignment of these signals may help not only to elucidate details of the biological nature of *E. coli* cell growth but also to develop the *E. coli* in-cell NMR methodology, enabling the elimination or filtering of undesired signals. Overexpression of the GB1 protein was started by adding isopropyl β-D-1-thiogalactopyranoside (IPTG) at OD_{600} = 1.80 to appear the unknown signals, and its 3D TROSY-HNCACB spectrum was measured (Supplemental Fig. S13). Its spectrum indicated that two of the unknown signals were derived from glycine residues (Supplemental Fig. S13A). However, although correlation signals between inter-residual Cα(i-1) and Cα(i) of glycine-candidate appeared in the in-cell 3D TROSY-HNCACB spectrum (Supplemental Fig. S13A), the origin of the inter-residue (i-1) correlation signal could not be assigned due to the corresponding signals being missing.

This assumption was further supported from the results of the glycine-selective inverse isotope labeling experiments and glycine-selective ^{1}H-^{15}N HSQC measurements using the MUSIC pulse scheme (G-HSQC) (Schubert et al. 1999; Schubert et al. 2001) (Supplemental Figs. S13B and S13C). In the glycine-selectively ^{14}N-labeled sample, the signal intensity of those unknown signals decreased significantly compared to that of a uniformly ^{15}N-labeled sample (Supplemental Fig. S13B). Furthermore, the G-HSQC

spectrum also provided support that the unknown first signal was certainly glycine (Supplemental Fig. S13C). The second of the two glycine-candidate signals (unknown signal No. 2) could not be observed in the G-HSQC due to weak signal intensity. From these results, it was assumed that the appearance of several unknown and undesired in-cell NMR signals could be suppressed as far as possible by glycine-selective isotopic unlabeling or coherence filtering.

It is known that D-alanine in a peptidoglycan of *E. coli* (denoted as red circles on the left panel of Supplemental Fig. S13D) can be replaced by glycine (Vollmer et al. 2008). In addition, the chemical shift of the inter-residual Cα(*i*-1) correlation signals of the HNCACB of the candidate of glycine was close to the Cα of *m*-A_2pm, a component of the cross-linking region between the glycan chains in a peptidoglycan of the *E. coli*; it was in the (*i*-1) position of the D-alanine (Supplemental Fig. S13D). These results suggest that the glycine-candidate unknown signals were derived from peptide fragments containing *m*-A_2pm–Gly bis-amino acid, generated from peptidoglycan of the *E. coli* host cells in a late phase of the cell growth.

In this study, we demonstrated the possibility that unknown NMR signals appearing uniquely in the *E. coli* in-cell NMR measurements were derived from peptidoglycan fragments containing glycine residue. However, in order to make definite identifications of the origin molecules in the future, further experiments using other techniques such as solid-state NMR are needed as there are many difficulties and limitations to the solution NMR approach in analyzing peptidoglycan, a large molecular assembly, even when fragmented.

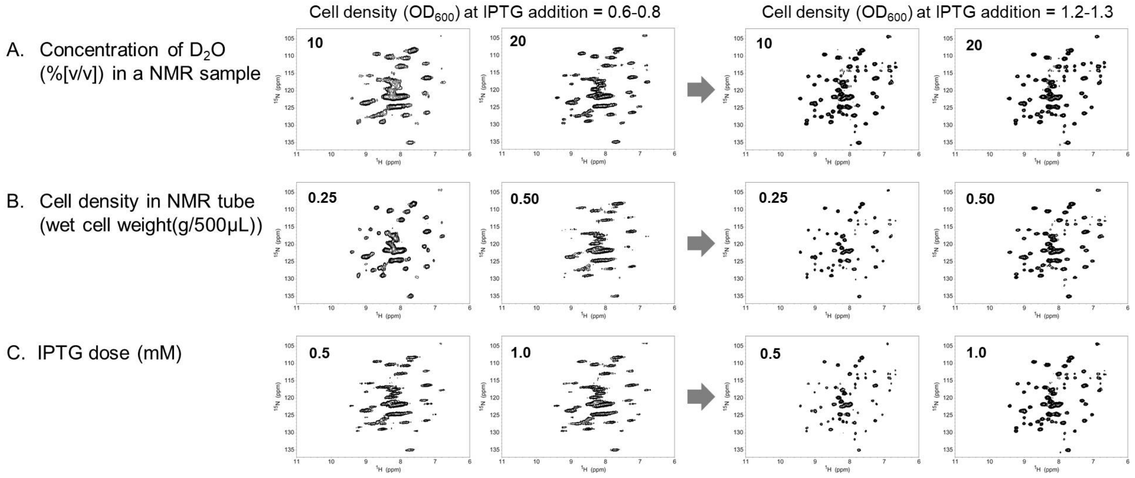

Supplemental Figure S1. Investigation comparing the experimental parameters for *E. coli* in-cell NMR measurements, namely the D_2O concentration of the NMR sample (A), the cell density in NMR sample (B), and dose of IPTG (C). The individual values of each parameter are indicated at the top left corner of each NMR spectrum. The cell density values (OD_{600} value) at the time of the addition of IPTG were 0.6-0.8 and 1.2-1.3 for left- and right-half six panels shown in left and right sides of arrows, respectively.

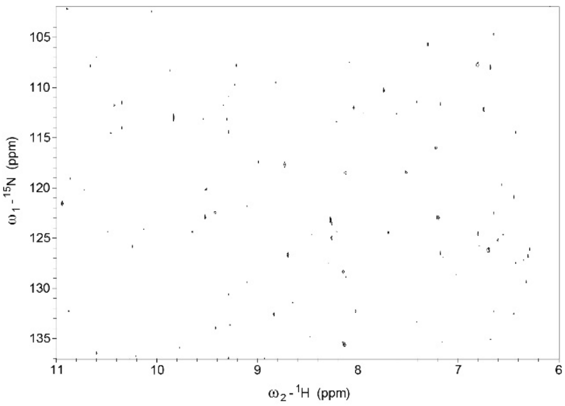

Supplemental Figure S2. The 2D ^1H-^{15}N TROSY-HSQC NMR spectrum of the supernatant of *E. coli* suspension immediately after the in-cell NMR measurement. The experimental conditions of the in-cell NMR experiments were identical to those of the NMR spectrum shown at the bottom right of Figure 1 (the OD_{600} value at the time of the addition of IPTG = 1.20; duration of the protein overexpression induction = 3 h).

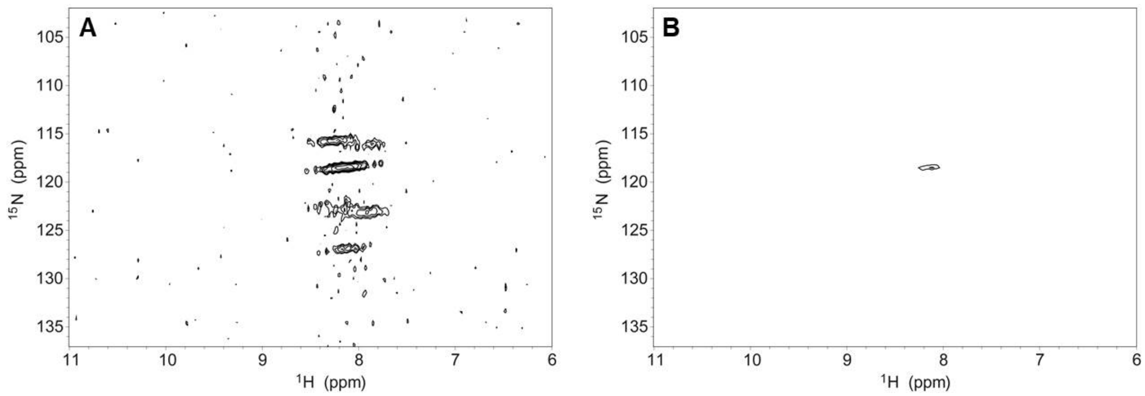

Supplemental Figure S3. The *E. coli* in-cell 2D ^{1}H-^{15}N TROSY-HSQC NMR spectra without induction of over-expression of the GB1 (pET) protein by IPTG. The contour levels are set to the lowest (A) and the same as Supplemental Figure S4 (B). These spectra show that the leakage expression from the pET expression plasmid is negligible.

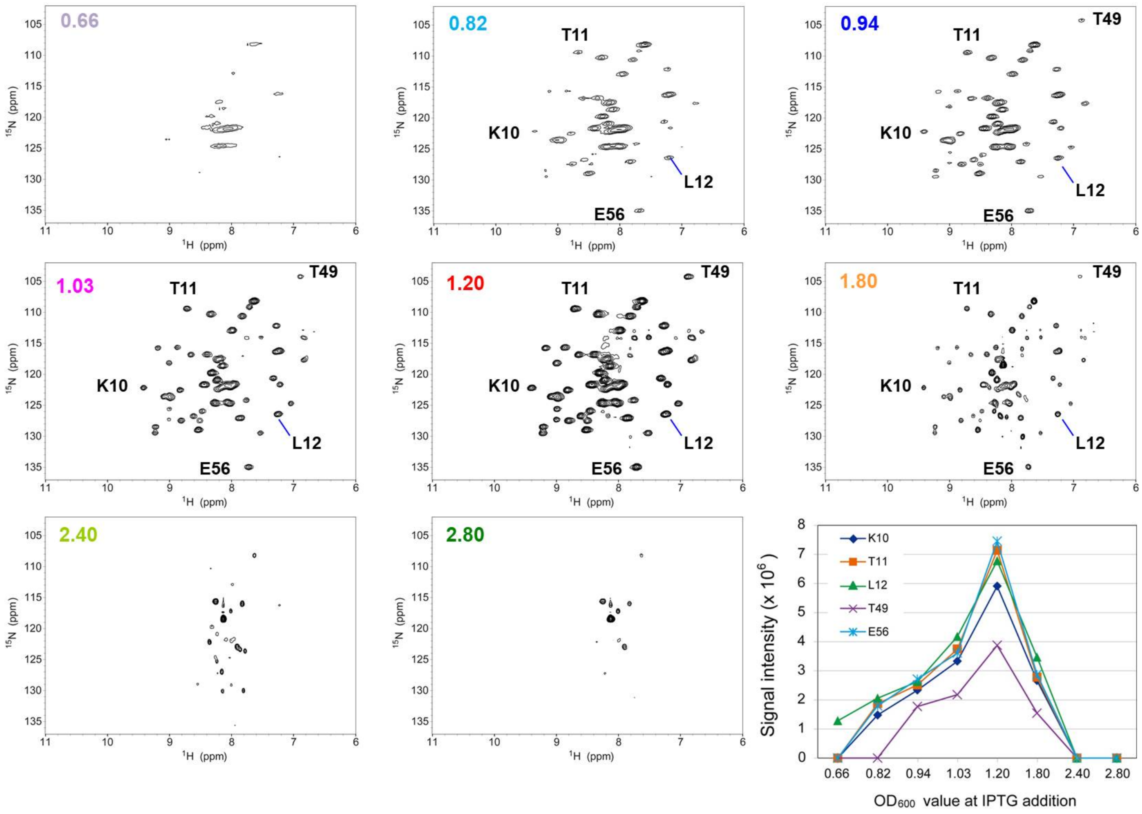

Supplemental Figure S4. *E. coli* in-cell 2D ^{1}H-^{15}N TROSY-HSQC NMR spectra for [*U*-^{15}N]GB1, same as Figure 1 except that the absolute values of the contour levels are set to identical for all spectra. Intensities of K10, T11, L12, T49, and E56 signals are plotted against the timing of IPTG addition (the bottom right panel).

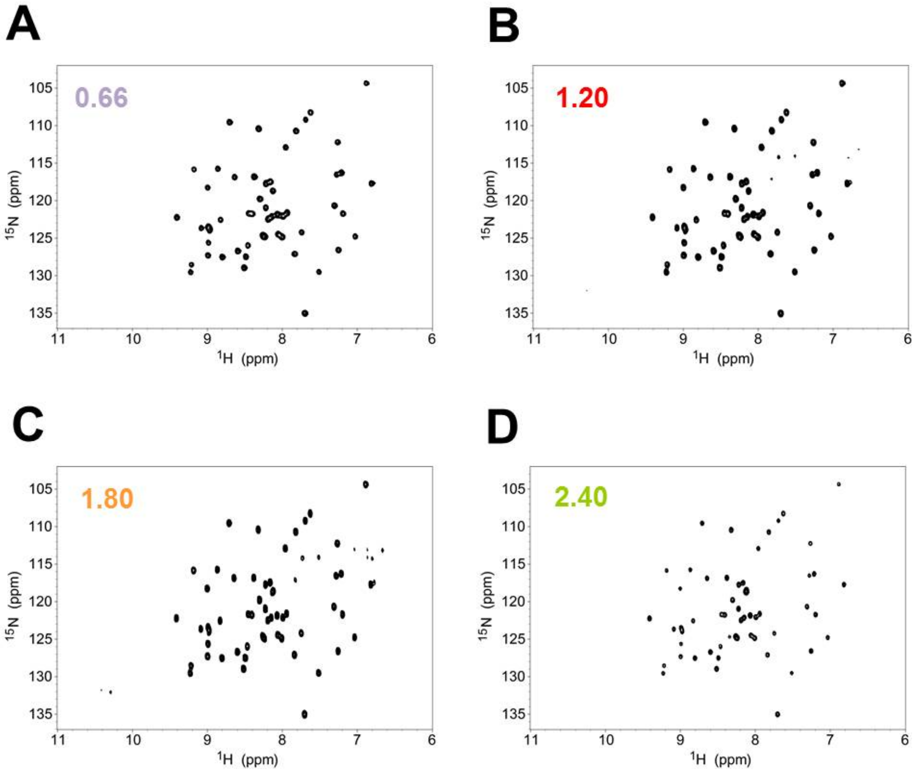

Supplemental Figure S5. A series of 2D ^{1}H-^{15}N TROSY-HSQC NMR spectra for [*U*-^{15}N]GB1 in the *E. coli* lysate. The lysate samples were prepared as follows; overexpression of [*U*-^{15}N]GB1 proteins were induced by adding IPTG into the *E.coli* cell culture at OD_{600} values of 0.66(A), 1.20 (B), 1.80 (C), and 2.40 (D), followed by 3 h cultivation, and then the *E. coli* cell pellet (ca. 0.55 g wet cell weight) was re-suspended with ~500 µL of fresh M9 minimal media containing 10% [v/v] D_2O as described in Materials and Methods section in the main text. Then, the cells were disrupted by performing sonication on ice for 3 min with a duty cycle of 1 s and 5 s off. The NMR spectra of the lysate were measured after cell debris was eliminated by centrifugation at 12,000 *g* for 10 min at 4 °C (Xu et al. 2014). The OD_{600} value at the timing of the addition of IPTG is indicated at the top left corner of each NMR spectra.

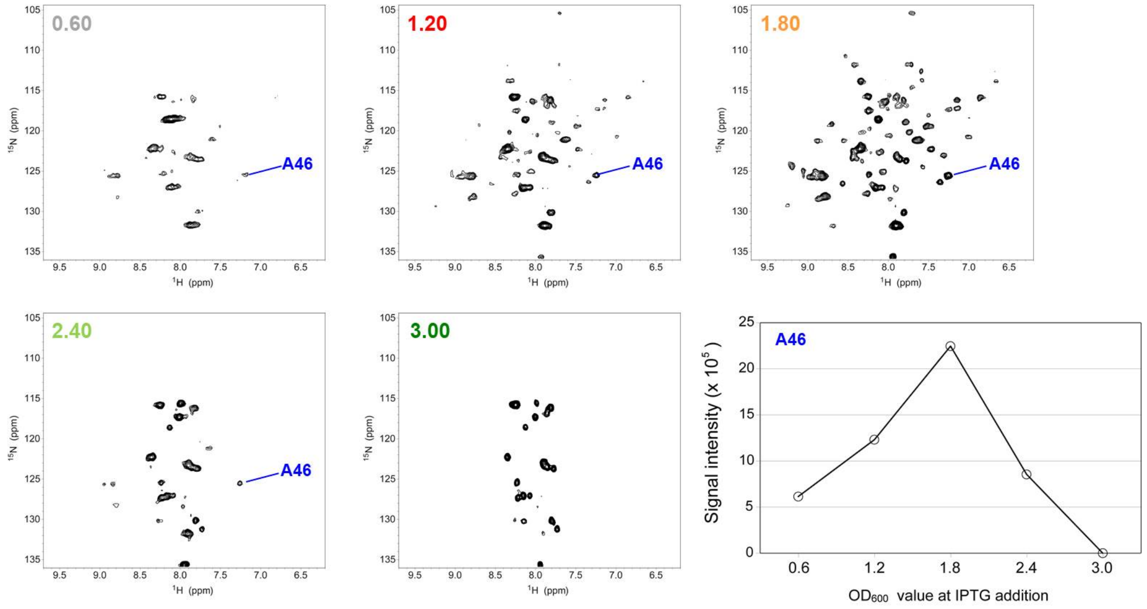

Supplemental Figure S6. *E. coli* in-cell 2D ^{1}H-^{15}N TROSY-HSQC NMR spectra for [*U*-^{15}N]TTHA1718, same as Figure 2 except that the absolute values of the contour levels are set to identical for all spectra. Intensities of A46 signals are plotted against the timing of IPTG addition (the bottom right panel).

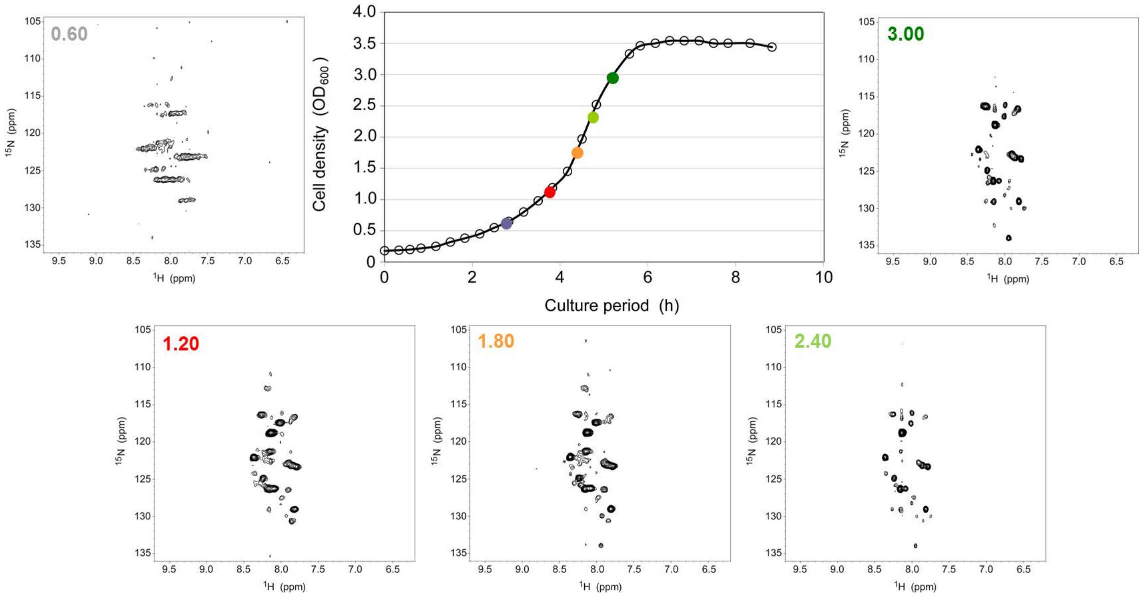

Supplemental Figure S7. A series of *E. coli* in-cell 2D ^{1}H-^{15}N TROSY-HSQC NMR spectra for [*U*-^{15}N]TTHA0227 were measured for various cell density (OD$_{600}$) values at the time of the addition IPTG. The growth curve for the pET11/TTHA0227 transformants of the *E. coli* BL21(DE3) strain is shown in the upper right panel. The OD$_{600}$ value at the time of the addition of IPTG to the individual in-cell NMR spectrum is denoted in the top left corner of each NMR spectrum.

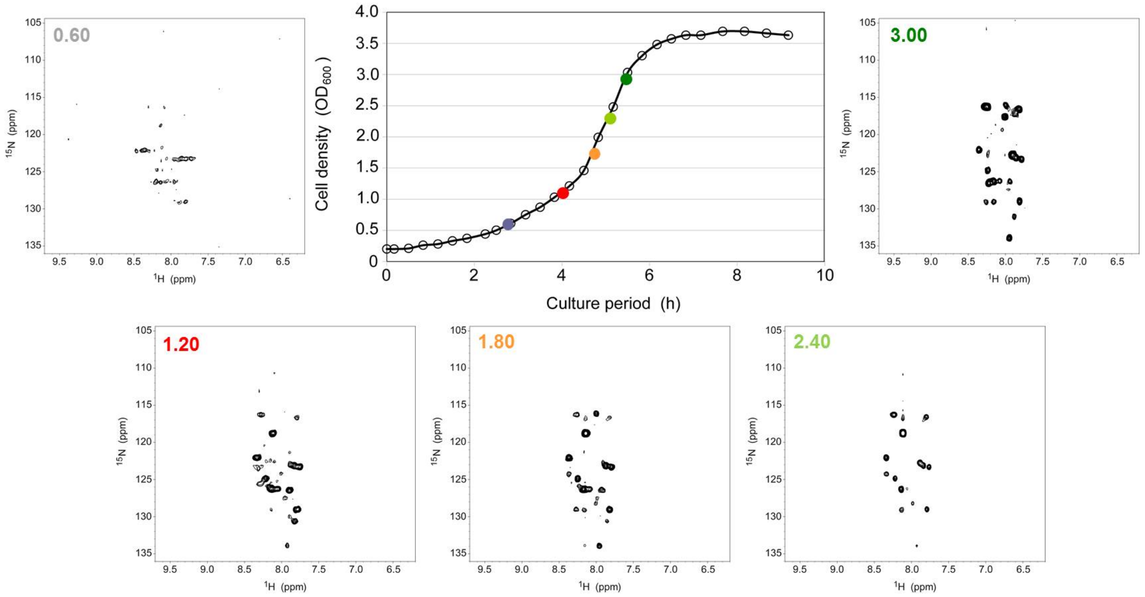

Supplemental Figure S8. A series of *E. coli* in-cell 2D ^{1}H-^{15}N TROSY-HSQC NMR spectra for [*U*-^{15}N]TTHA0814 were measured for various cell density (OD_{600}) values at the time of the addition of IPTG.The growth curve for the pET11/TTHA0814 transformants of the *E. coli* BL21(DE3) strain is shown in the upper right panel. The OD_{600} value at the time of the addition of IPTG to the individual in-cell NMR spectrum is denoted in the top left corner of each NMR spectrum.

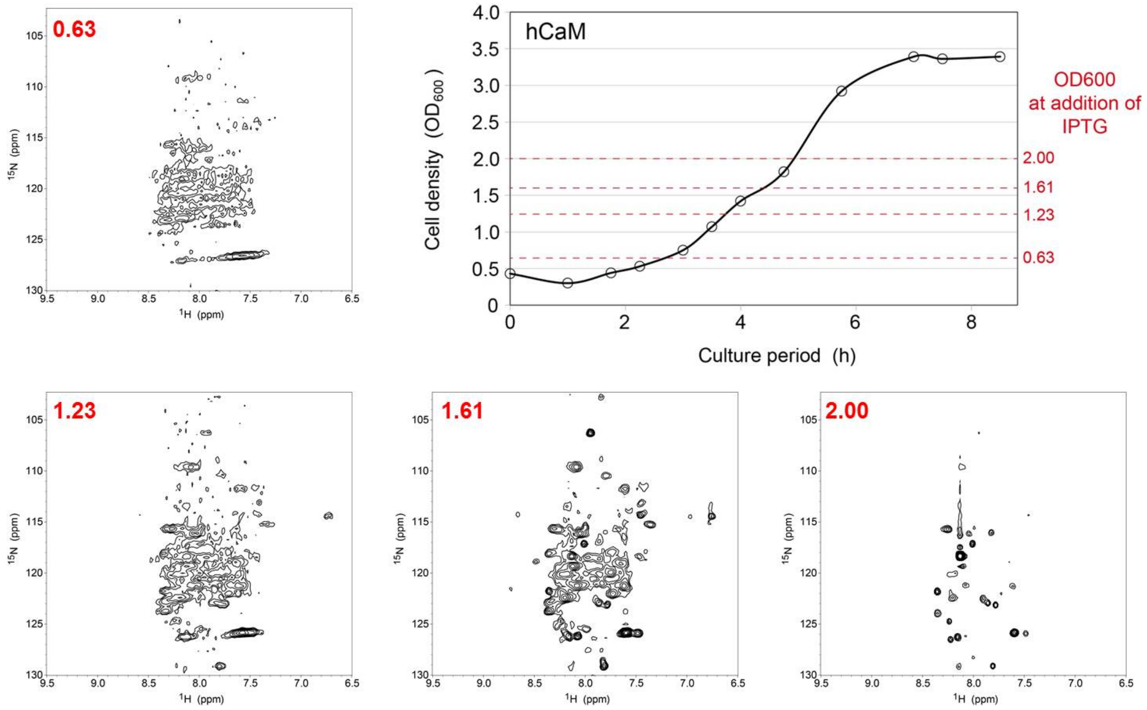

Supplemental Figure S9. A series of *E. coli* in-cell 2D ^{1}H-^{15}N TROSY-HSQC NMR spectra for [*U*-^{15}N]hCaM were measured for various cell density (OD_{600}) values at the time of the addition of IPTG. The growth curve for the pET21/hCaM transformants of the *E. coli* BL21(DE3) strain is shown in the upper right panel. The OD_{600} value at the time of the addition of IPTG to the individual in-cell NMR spectrum is denoted in the top left corner of each NMR spectrum.

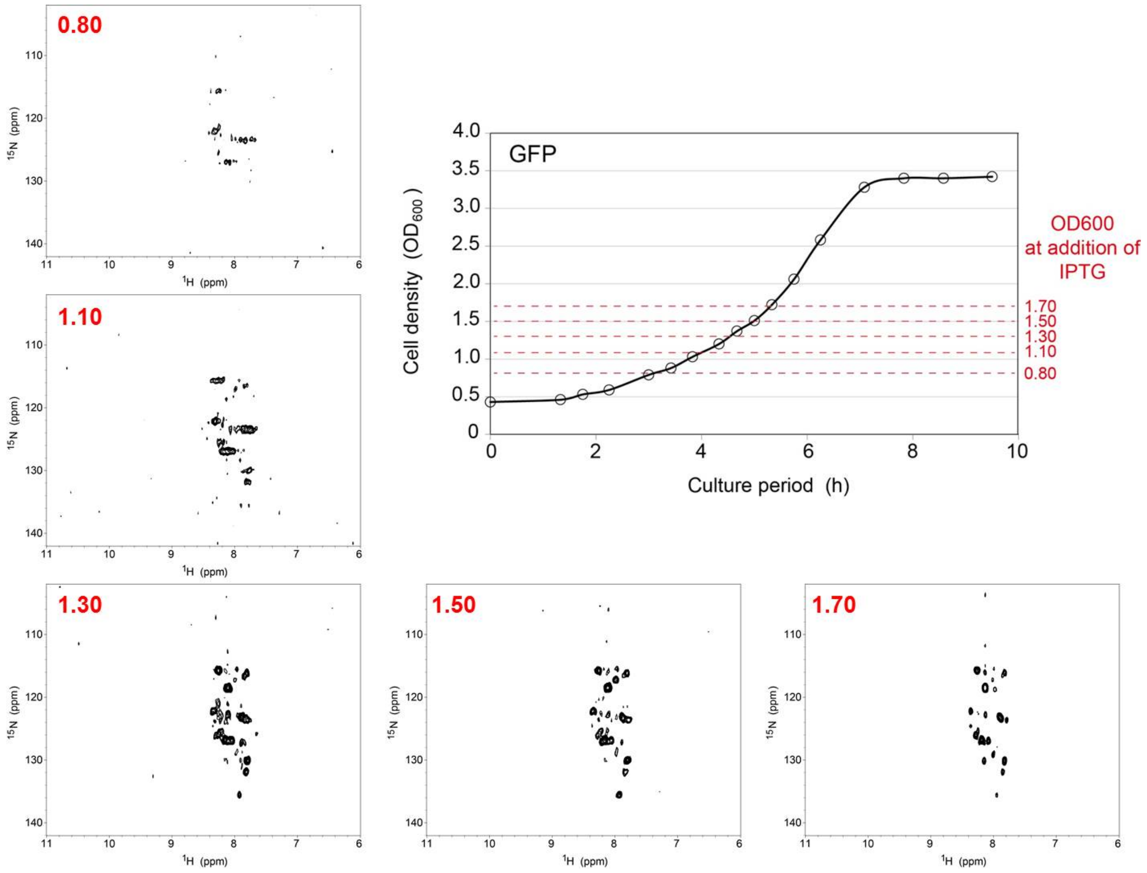

Supplemental Figure S10. A series of *E. coli* in-cell 2D ^{1}H-^{15}N TROSY-HSQC NMR spectra of [*U*-^{15}N] GFP measured at varying cell densities (OD_{600} values) at the time of the addition of IPTG. The growth curve of the GFP transformants of the *E. coli* BL21(DE3) strain is shown in the upper right panel. The OD_{600} value at the time of the addition of IPTG for each individual in-cell NMR spectrum is indicated on the top left corner of the spectrum.

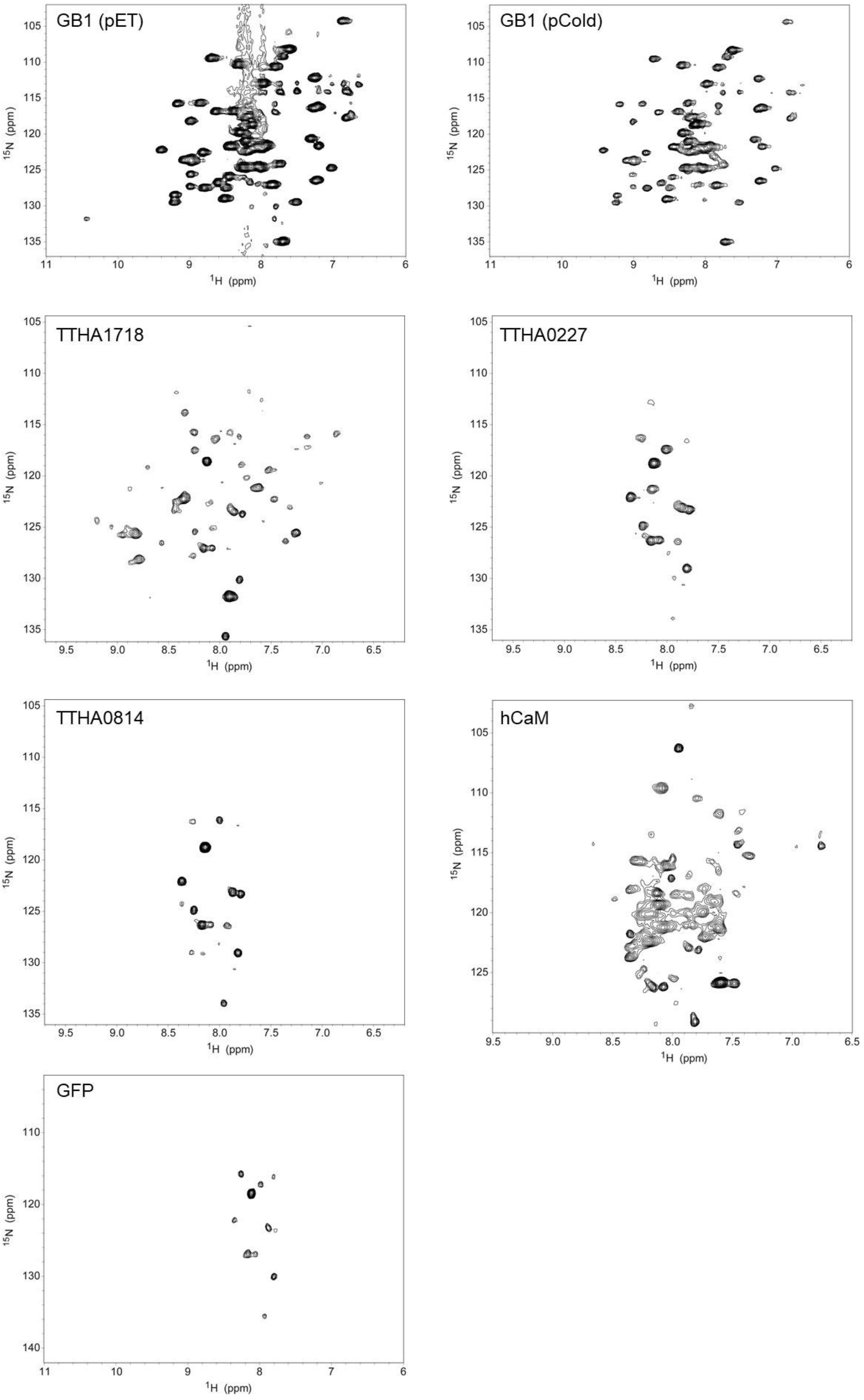
GB1 (pET)
GB1 (pCold)
TTHA1718
TTHA0227
TTHA0814
hCaM
GFP
15N (ppm)
1H (ppm)

Supplemental Figure S11. *E. coli* in-cell 2D ^{1}H-^{15}N TROSY-HSQC NMR spectra for all proteins induced at the early to middle log phase. The cell densities (OD_{600} values) at the time of the addition of IPTG for overexpression of GB1 (pET system), GB1 (pCold system), TTHA1718, TTHA0227, TTHA0814, hCaM, and GFP are 1,20, 1.00, 1.80, 1.80, 1.80, 1.61, and 1.50, respectively. The absolute values of the contour levels are set to identical for all spectra.

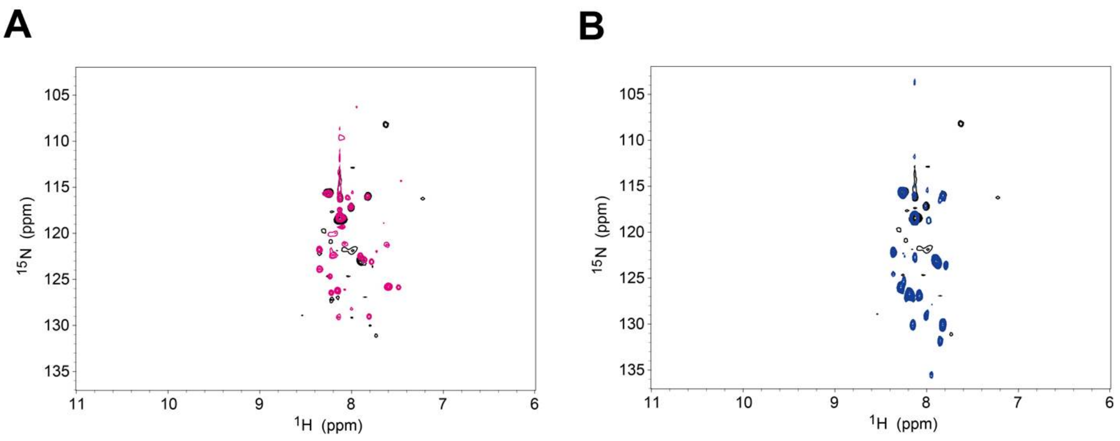

Supplemental Figure S12. An overlay of the *E. coli* in-cell 2D ^{1}H-^{15}N TROSY-HSQC NMR spectra. Black, GB1 (the OD_{600} value at the time of the addition of IPTG = 2.80). (A) Magenta, hCaM (the OD_{600} value at the time of the addition of IPTG = 2.80). (B) Blue, GFP (the OD_{600} value at the time of the addition of IPTG = 1.70)

A

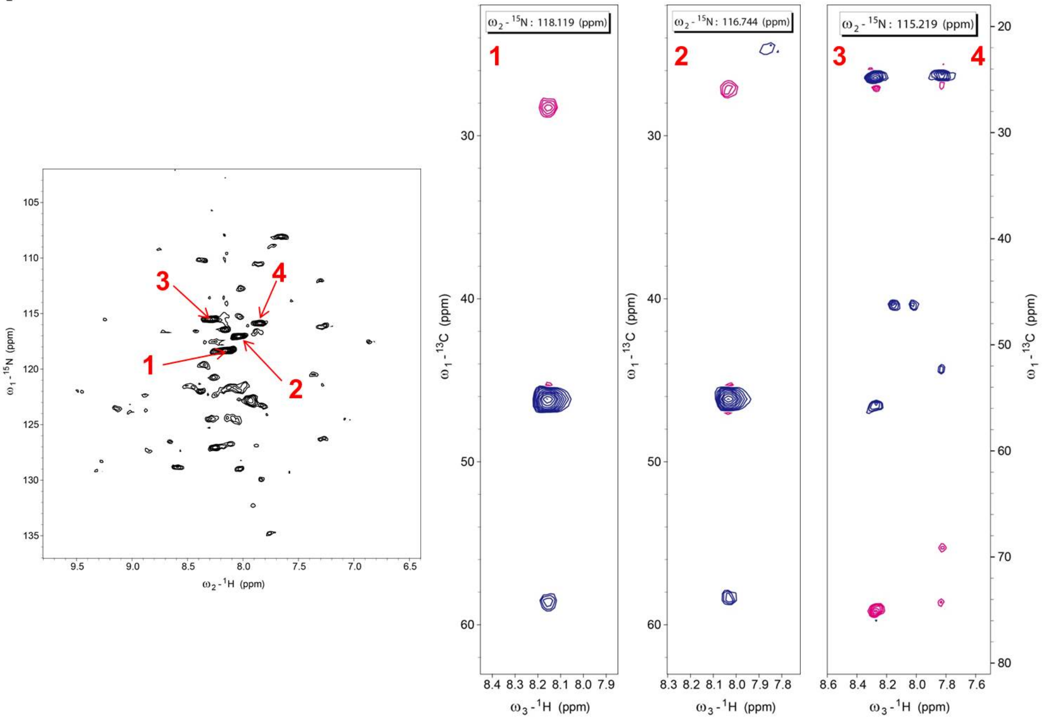

Supplemental Figure S13 (continued)

B

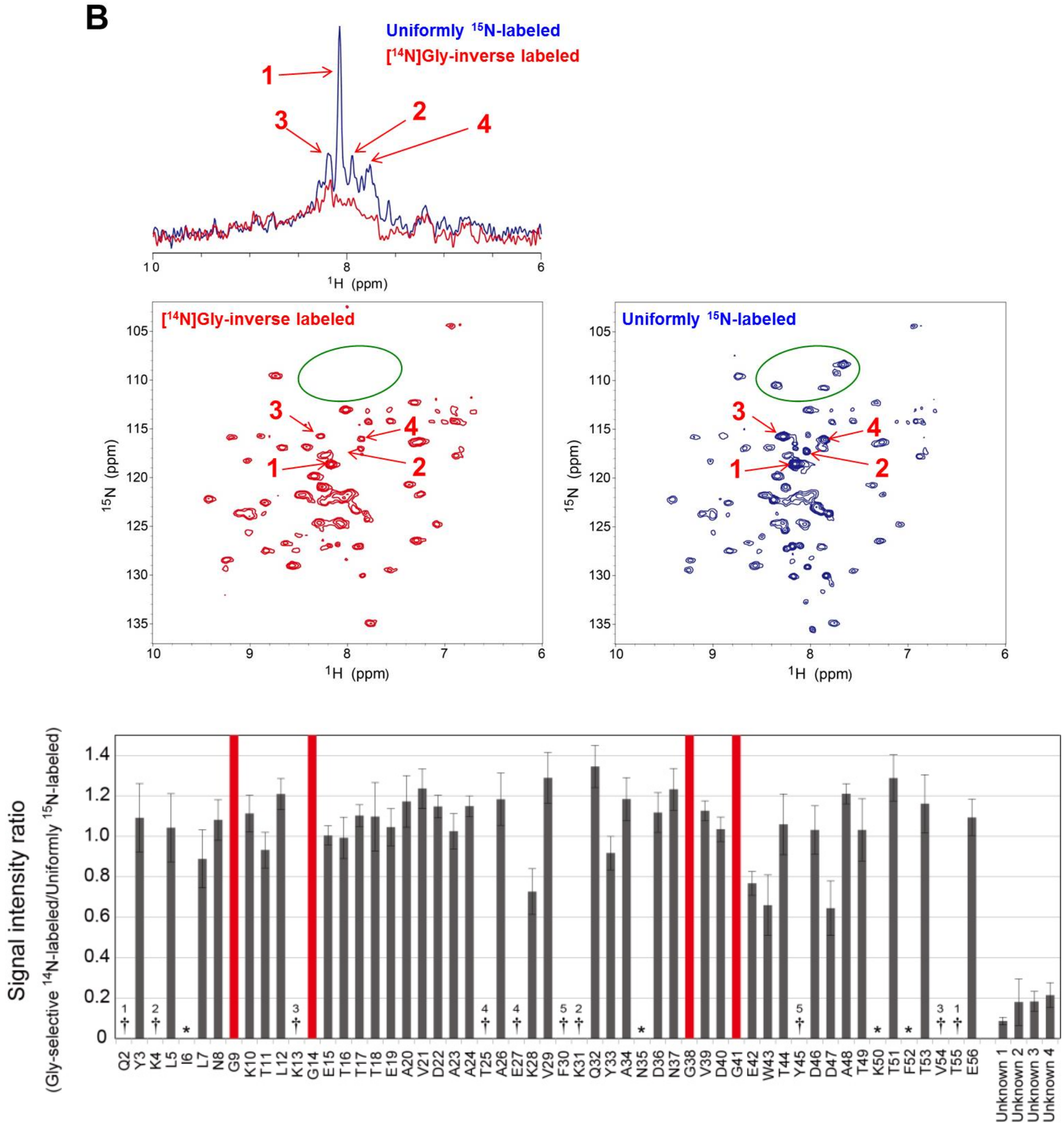

Supplemental Figure S13 (continued)

C

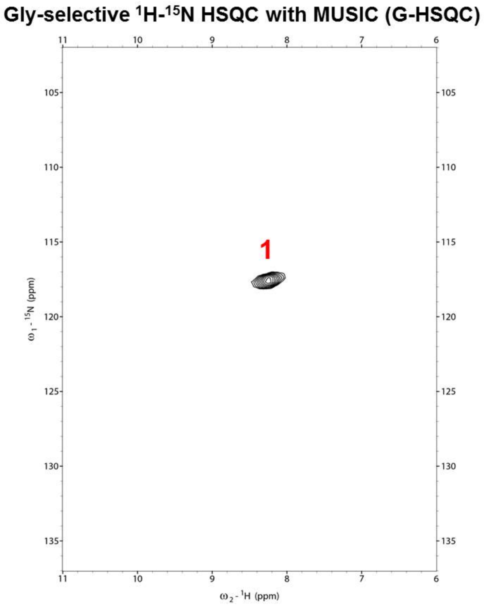

D

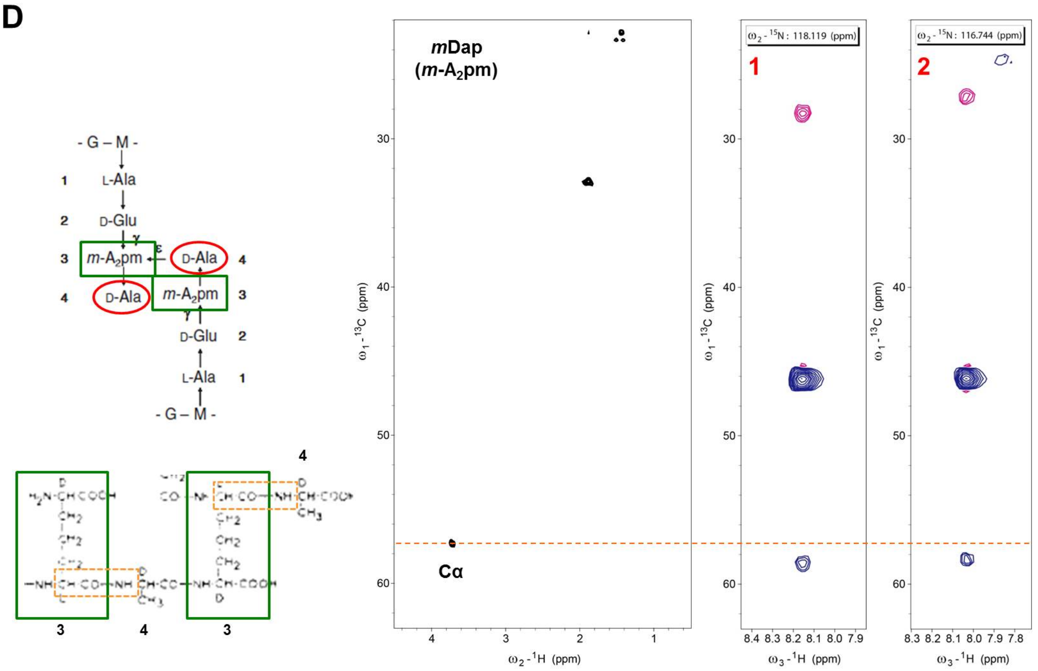

Supplemental Figure S13. The identification of significant unknown signals that appeared when protein overexpression induction was started during the middle or late logarithmic cell growth phases (OD_{600} > 1.20). (A) 2D ^{1}H-^{15}N TROSY-HSQC (left panel) and 2D $^{1}H^{N}$-$^{13}C^{\alpha,\beta}$ strips of the 3D TROSY-HNCACB spectra (right panels) for

the unidentified signals. Blue and magenta indicate positive and negative signs, respectively. The red numbering on the spectra indicates each unknown signal. (B) Upper panels: 1D slices and 2D spectra of ^{1}H-^{15}N TROSY-HSQC of glycine (Gly)-selectively inverse (^{14}N)-labeled (red) and uniformly ^{15}N-labeled (blue) GB1. The green open circles on these 2D spectra indicate the signals of four glycine residues of GB1 (Gly9, Gly14, Gly38, and Gly41). Lower panel: Plots of signal intensity ratio of Gly-selectively inverse-labeled GB1 versus uniformly ^{15}N-labeled GB1. The red bars show that the signals of all glycine residues of GB1 (Gly9, Gly14, Gly38, and Gly41) had completely disappeared by the Gly-selective inverse labeling as denoted by the green open circle on the 2D ^{1}H-^{15}N TROSY-HSQC spectra. The obelisks indicate overlapping signals between Q2 and T55 (†1), K4 and K31 (†2), K13 and V54 (†3), T25 and E27 (†4), F30 and Y45 (†5). Asterisks indicate missing signals. (C) In-cell Gly-selective ^{1}H-^{15}N HSQC with MUSIC (G-HSQC) of GB1. (D) Position of the *m*-A_2pm in a peptidoglycan of *E. coli* (denoted as green open squares in the left panel) and a 2D ^{1}H-^{13}C HSQC spectrum of purified *m*-A_2pm (center panel). The 2D $^{1}H^{N}$-$^{13}C^{\alpha,\beta}$ strips of the 3D TROSY-HNCACB spectra of unknown signals Nos. 1 and 2 (right panels) are identical to those of Supplemental Figure S13A.

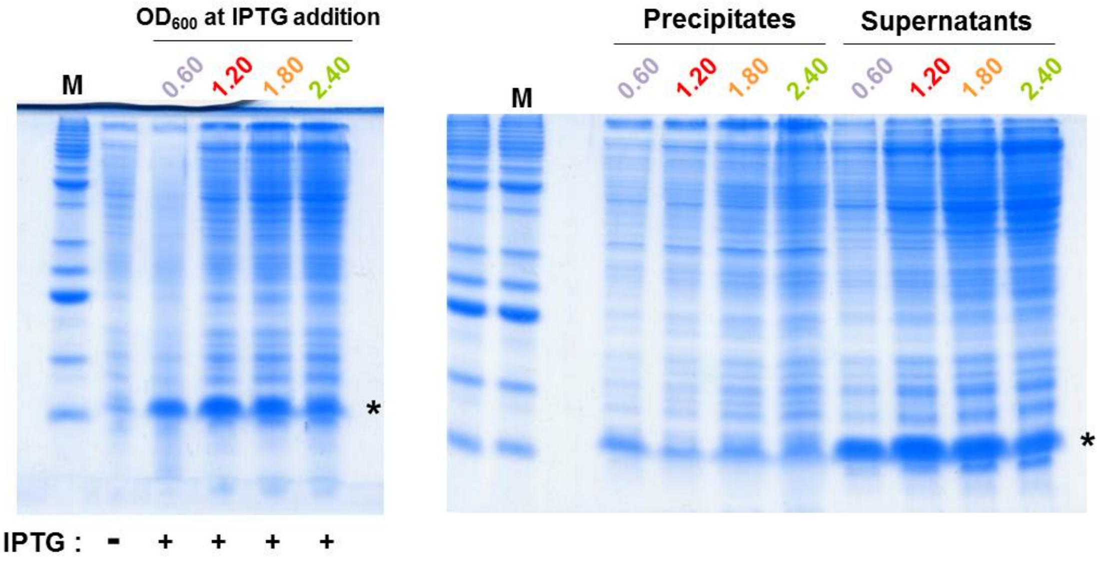

Supplemental Figure S14. The entire SDS–PAGE results corresponding to those shown in Figure 1. The left panel shows the whole protein expression level and its dependency on the OD_{600} value at the addition of IPTG. The asterisks indicate the bands of the overexpressed heterologous target protein (GB1).

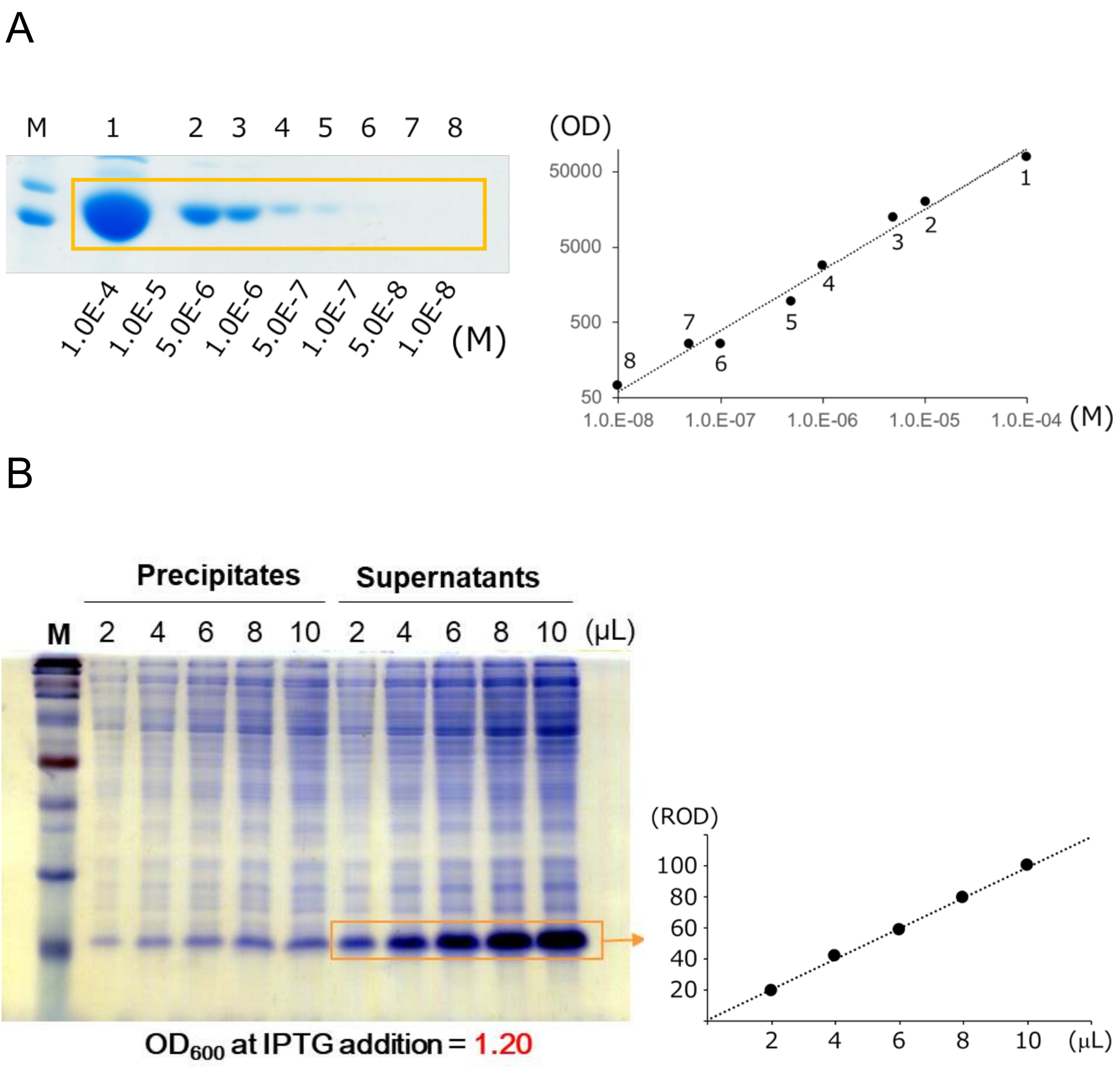

Supplemental Figure S15. (A) Densitometry of SDS-PAGE bands calibrated for a purified protein, 24 kDa MSP1D1. The linear regime is 10^{-4} ~ 10^{-8} M in our system employing the flatbed scanner and the software ImageJ. (B) Densitometry of SDS-PAGE bands for GB1 corresponding to those shown in Figure 1 for the OD_{600} value at the time of the addition of IPTG = 1.2. The entire SDS-PAGE results are shown changing the loaded sample volume within a same gel. ROD is relative optical density assuming 10 μL sample as 100%. The loaded sample volume for the SDS-PAGE shown in Figure 1 is 10 μL.

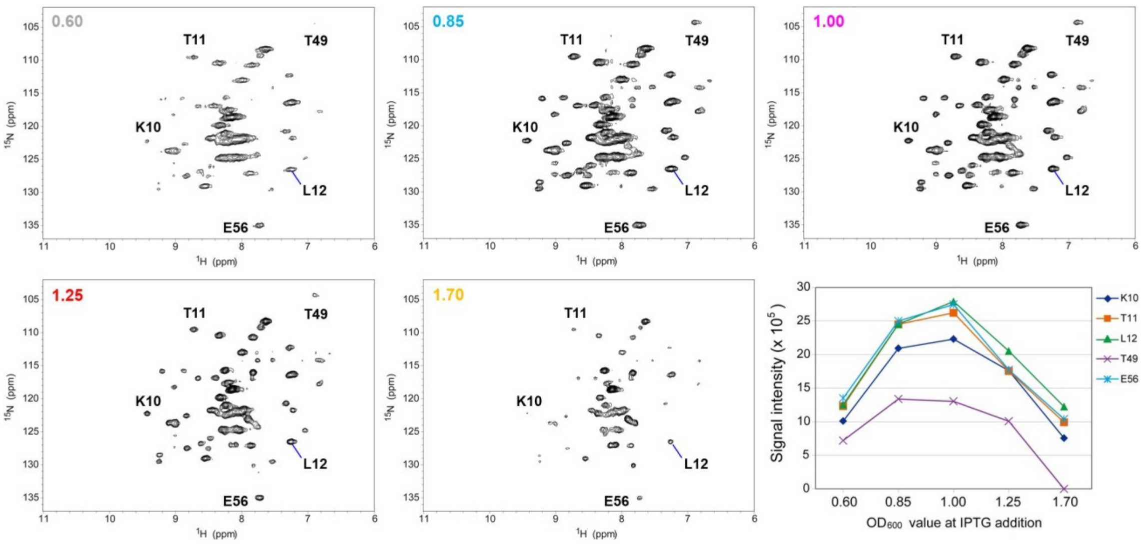

Supplemental Figure S16. *E. coli* in-cell 2D ^{1}H-^{15}N TROSY-HSQC NMR spectra for [*U*-^{15}N]GB1 with the protein overexpressed by using the pCold expression system, same as Figure 3, except that the absolute values of the contour levels are set to identical for all spectra. Intensities of K10, T11, L12, T49, and E56 signals are plotted against the timing of IPTG addition (the bottom right panel).

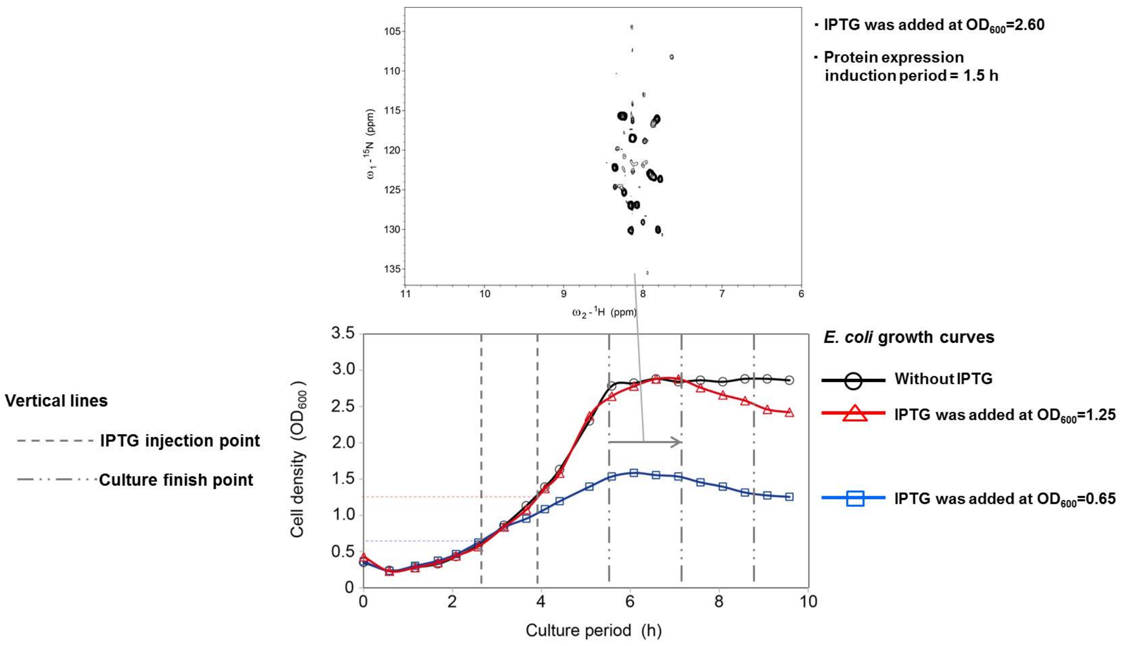

Supplemental Figure S17. An *E. coli* in-cell 2D ^{1}H-^{15}N TROSY-HSQC NMR spectrum corresponding to the gray arrow drawn on the growth curve panel (the OD_{600} value at the time of the addition of IPTG = 2.60; duration of the overexpression of target protein induction = 1.5 h). This growth curve is identical to that of Figure 4.

References

Serber, Z., Keatinge-Clay, A. T., Ledwidge, R., Kelly, A. E., Miller, S. M., and Dötsch, V. (2001a) High-resolution macromolecular NMR spectroscopy inside living cells. *J. Am. Chem. Soc.* ***123***, 2446–2447

Serber, Z., Ledwidge, R., Miller, S. M., and Dötsch, V. (2001b) Evaluation of parameters critical to observing proteins inside living *Escherichia coli* by In-Cell NMR spectroscopy. *J. Am. Chem. Soc.* ***123***, 8895–8901

Schubert, M., Smalla, M., Schmieder, P., and Oschkinat, H. (1999) MUSIC in triple-resonance experiments: amino acid type-selective ^{1}H-^{15}N correlations. *J. Magn. Reson.* ***141***, 34–43

Schubert, M., Oschkinat, H., and Schmieder, P. (2001) MUSIC, selective pulses, and tuned delays: amino acid type-selective ^{1}H-^{15}N correlations, II, *J. Magn. Reson.* ***148***, 61-72

Vollmer, W., Blanot, D., and de Pedro, M.A. (2008) Peptidoglycan structure and architecture. *FEMS Microbiol. Rev.* ***32***, 149-167

Xu, G., Ye, Y., Liu, X., Cao, S., Wu, Q., Cheng, K., Liu, M., Pielak, G.J., and Li, C. (2014) Strategies for protein NMR in *Escherichia coli*. *Biochemistry* ***53***, 1971-1981
